# Wearable device for tremor-like motion acquisition: a low-cost device using 3D printing for research and education

**DOI:** 10.1016/j.ohx.2026.e00823

**Published:** 2026-08-06

**Authors:** Nhut Thang Le, Ngoc Hung Nguyen, Cong Toai Truong, Huy Hung Nguyen, Tan Tien Nguyen, Van Tu Duong

**Affiliations:** a Key Laboratory of Digital Control and System Engineering (DCSELab), Faculty of Mechanical Engineering, Ho Chi Minh University of Technology (HCMUT), 268 Ly Thuong Kiet Street, Dien Hong Ward, Ho Chi Minh City, Viet Nam; b Vietnam National University Ho Chi Minh City, Linh Xuan Ward, Ho Chi Minh City, Viet Nam; c Faculty of Electrical and Electronics Engineering, Ho Chi Minh City University of Technology and Engineering, Ho Chi Minh City, Vietnam

**Keywords:** Parkinson’s disease, Tremor monitoring, Wearable device, 3D printing, Low-cost, Open-source hardware

## Abstract

Parkinson’s disease (PD) is a growing neurodegenerative disorder, with increasing global prevalence and challenges in both diagnosis and education. To assist in researching the domain of medical equipment and education training, this paper presents the design and implementation of a wearable device for continuous tremor-like motion monitoring in a laboratory prototype setting using low-cost materials and easy-to-build hardware. The tremor-like motion acquisition (TMA) is designed to be lightweight and non-intrusive, providing real-time acceleration-based motion measurement on the upper limb by measuring acceleration for both engineering research and educational demonstration. Notably, the TMA is constructed using fused deposition modeling techniques, offering a cost-efficient and rapid prototyping solution. Powered by a rechargeable battery, the TMA is designed for portability, with modular components that ensure flexibility and ease of maintenance. In addition, experimental tests are performed with a slider-crank mechanism to evaluate the data acquisition and motion-tracking capability under controlled mechanical excitation. As a result, the TMA meets design criteria, consisting of being lightweight, approximately 115 g, and low-cost, with a total hardware cost of $106.17, with a fast manufacturing time of 10 h. Furthermore, the monitoring tremor algorithm operates with results following the motion of the slider-crank mechanism and feeds the real-time data to software designed for smartphones, and no human-subject or clinical validation is conducted in this study.

## Graphical abstract

**Figure:**
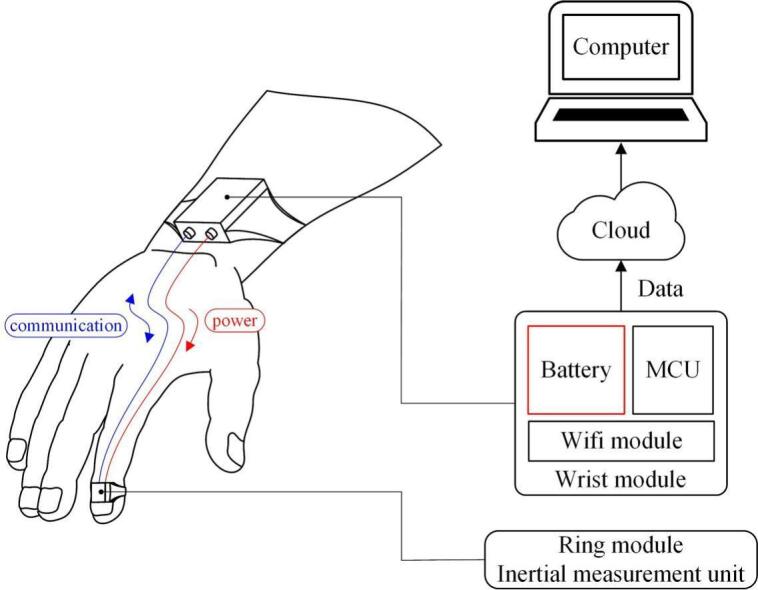

**Specifications table**

**Table:** 

Hardware name	Tremor-like motion acquisition device
Subject area	• Educational tools and open-source alternatives to existing infrastructure • General
Hardware type	• Field measurement and sensors
Closest commercial analog	PDMonitor, Surface electromyography devices.
Open-source license	CC BY 4.0
Cost of hardware	$106.17
Source file repository	https://doi.org/10.17632/859c935kb8.2

## 1 Hardware in context

In modern healthcare, biomedical devices [1], [2], [3], model identification [4], [5], [6], simulation systems [7], and lightweight actuator control solutions [8], [9], [10] have been widely developed across miscellaneous fields, serving as fundamental building blocks for creating therapeutic tools tailored to specific clinical needs. In recent years, Parkinson’s disease (PD) and neurodegenerative disorders have emerged as long-standing yet increasingly prevalent conditions that significantly impact patients’ health and quality of life. In reality, PD represents one of the fastest-growing neurodegenerative disorders globally, with the increasing trend to an estimated 9.4 million cases in 2023 and projected to double by 2050 due to demographic aging [11], [12]. Demand to [13], [14], the approach to managing PD depends on the neurologist’s experience and the trustworthiness of the reported symptom information. Unfortunately, symptom reporting is discontinuous since the symptoms of PD can occur at any time without warning. Therefore, it may happen that the patients fail to document their mild or changing symptoms, while the clinicians evaluate these symptoms during regular clinic appointments [15], [16], [17]. Especially, minor changes in motor functions can escape notice as well, particularly if the changes fail to influence the daily activity level of a person [18]. In practice, for developing doctors who are going to diagnose early-stage Parkinson’s disease through subtle movement problems such as bradykinesia, there is a need to improve medical training so that medical students have training using real-life movement data from patients [19]. However, based on findings from the literature survey, it appears that there is a gap in education, whereby there is a lack of a tremor monitor device that can make use of quantified data for students. This means that there are quite rare devices which can be used to make theoretical knowledge practical, hence difficult to detect motor dysfunctions from early onset to advanced stages [20]. From there, this limitation drives the development of user-friendly motion-sensing systems that enable the analysis and visualization of movement data for educational purposes in research training, engineering education, and preliminary algorithm development.

In an effort to solve this problem, several types of equipment that have been commercialized, as well as some research methods, have been proposed to collect and study data on the tremors exhibited by the patients. One such device is the PDMonitor™, which consists of five different types of equipment that help identify all motor symptoms of a PD patient [21], [22]. Although its machine learning algorithms, which have been trained using expert-labeled data, show good correlation with UPDRS scores and patient logs, it faces certain limitations that make its practical implementation difficult. The multi-layered nature of the device makes putting it on difficult, and its bulky appearance may lead to non-compliance because of embarrassment. Additionally, the high cost, which is approximately $11,000, is an economic constraint, preventing the access of the devices by individuals living in low-developed or developing nations. Furthermore, smartwatches and various wearable devices equipped with multiple sensors for their different functionalities are discussed in relation to HAR application in medicine, behavior, and gesture recognition [23]. Devices utilizing Apple Watch technology are notable examples, designed using proprietary software running on the Apple Watch capable of tracking dyskinesias and tremors [24], [25]. Unfortunately, the algorithms used lack transparent, and their high prices of more than $400 make it impossible to use them widely. These devices tend to suffer from restricted use because they can continuously and objectively measure the progression of diseases that would allow neurologists to intervene according to the results of longitudinal ambulatory monitoring. Apart from privately owned monitoring devices, EMG devices [26], such as Cadwell EMG Sierra Summit, are commonly used in hospitals for diagnosing and monitoring patients with Parkinson’s disease. These devices provide comprehensive EMG graphical outputs that help healthcare professionals evaluate the state of their patients. Nevertheless, considering the high cost of the device amounting to $14,000 for each unit along with costly treatment sessions, these fail to affordable. This leads to an inability to make a record of the information continuously throughout the day; hence, failing to capture the results of the full medical examination. Overall, there still remain major issues preventing the implementation of PD monitoring devices commercially. First, the issue is how reliable and precise the devices are, given the features that characterize automated analysis. Secondly, there is a lack of functionality for Parkinson’s disease measurements in the currently available devices when it comes to motion tracking features. Lastly, the monitoring tools for PD that can be bought commercially cost thousands of dollars, making them too expensive for students studying neurological diseases.

Thus, there is an immediate need for affordable and reproducible sensing systems that allow students and researchers to acquire, visualize, and analyze tremor-like motion data under controlled conditions. In response, this research proposes a device that not only ensures data acquisition is constantly acquired without affecting the external disturbance but also affords students and researchers with both educational purposes and testing tremor data analyzing algorithms. Furthermore, with the simple and accessible design, the device can be easily manufactured at an affordable cost, allowing both underdeveloped and developing nations to produce and master their technology.

## 2 Hardware description

The Tremor-like motion acquisition device (TMA) is an ergonomic wearable designed to monitor the low-frequency vibration, which is tremor-like motion, in the under controlled conditions for both research training and engineering education. Constructed primarily from acrylonitrile butadiene styrene (ABS) via fused deposition modeling (FDM), the device leverages additive manufacturing advantages, including biocompatibility, cost efficiency, and rapid prototyping flexibility, through 3D-printed applications. From a biomedical standpoint, the proposed device’s enclosure is fabricated from ABS, a widely used material for wearable and external-contact devices due to its low weight and acceptable skin compatibility for non-invasive applications. The choice of materials complies with the overall guidelines suggested by the ISO 10993 standard on biological assessment of medical devices, which suggests that special attention be paid to avoiding irritation and sensitization for devices that will have contact with the skin [27]. The mechanical components, such as the microcontroller base, the mainboard base cover, the strap fixing, the ring base, and the ring cover, are printed via the FDM process, and the total printing time is about 10 h. The entire device weighs around 115 g, and the dimensions of the entire device are (30x55x60) mm, making it very lightweight and portable to be worn comfortably without restricting the motion of the hand, as well as suitable to be used even in laboratories and classrooms.

The mechanical assembly is characterized by its high level of modularity and user-friendly design. TMA consists of two major parts: the wrist-wear part and the finger-wear part. In the wrist-wear part, the mainboard, the microcontroller cover, and the strap fixing system are assembled and mounted on the wrist using a medical plastic strap. In the finger-wear part, the microcontroller cover is assembled and mounted on the ring using a ring strap. There are dedicated mounting slots in the microcontroller base and the ring for the installation of the mainboard and IMU sensors, respectively. The modular design helps make the maintenance process simple and easy. Therefore, the design of the device involves the use of a non-invasive, wearable design. This is achieved by connecting both the ring and wrist units using adjustable straps that are similar to ordinary watch straps. These straps allow for proper fixation of the device onto the arm without any penetration or interference with biological tissues. Moreover, the total weight of the device meets the ergonomic standards of wearable computers [28].

In terms of the electric point of view, the TMA has its source of power as the battery that can be purchased and charged. In the existing model, the use of a battery pack 10000 mAh 5VDC is adopted to provide power to the system constantly. Considering the electric components in TMA, the maximum current would be 1A. To determine the maximum constant operational time for TMA, Eq. (1) [29] is used:

(1) $$L=\frac{C}{I},$$

where C is the capacity of the battery, and I is the current drawn from it. Based on Eq. (1), the TMA can run for up to 10 h. However, to ensure the optimal functioning of the TMA and to avoid any incidence of voltage drop caused by loading during data transmission and IMU operation, a period of 8 h is recommended. The factor of safety enhances the life of the battery while ensuring the uniformity of the measurements throughout the process. A battery with greater capacity may be used if it is needed to extend the duration, but importantly, a 5VDC output has to be maintained. Linear LDO regulators are employed to regulate the voltage down from 5 V to 3.3 V for the MCU and IMUs attached to the circuit board. Although the linear regulator is less efficient than the buck converter, which makes use of the switched-mode power supplies, producing more heat, it was chosen due to its simplicity. Safety considerations from an electrical perspective show that the gadget utilizes a battery voltage power source of 5 V, and 5 V falls in the safe extra-low voltage (SELV) category, which is the recommended voltage for safe operation of electronic gadgets [30]. Additionally, the PCB mainboard comes with some protective elements, like filters through capacitors, fuses, and diodes, in order to counter any variations in voltage, hence enhancing its safety when in operation. Moreover, the board has some expandable communication headers together with serial communication ports, making it easy to add other components, such as Wi-Fi connections and closed-loop systems.

As a result, the contribution of the hardware TMA is comprised of:

• Offer a cost-effective wearable prototype fabricated using a fully 3D-printed FDM enclosure, enabling rapid manufacturing and easy reproduction.
• Features a modular wrist–finger architecture that supports flexible assembly, convenient maintenance, component replacement, and hardware expansion.
• Provide an open-source-compatible sensing platform integrating acceleration acquisition, embedded control, and real-time data transmission for engineering implementation.
• Supports an educational and research-oriented prototype that provides hands-on training in wearable sensing and provides a preliminary basis for future tremor-related motion studies.

## 3 Design files summary

### Design file descriptions

TMA_mainboard: comprises the schematic design and PCB layout of the main control board used in the TMA_PCB. It specifies components’ interconnections, signal routing, and power distribution, supporting system control and peripheral communication.

TMA_PCB: includes the complete Altium Designer project, encompassing the main TMA controller’s schematic and multi-layer PCB layout. It defines the functional structure of the board, component placements, track routing, and layer configuration, which ensures signal integrity and electrical performance during operation.

TMA_Assem: contains the 3D mechanical assembly model of the TMA, consisting of the printed circuit board, mechanical shell, and others. This assures the correct assembly of the components in their proper place.

TMA_Source_code: includes the firmware for the TMA controller, implementing all control algorithms for analyzing tremor data. It handles the configuration of tremor parameters and communication.

## 4 Bill of materials summary

(See Table 2, Table 3).

**Table 1:** List of components for the TMA.

**Design file name**	**File type**	**Version**	**Open-source license**	**Location of the file**
TMA_Mainboard	PDF	1.0	CC BY 4.0	https://doi.org/10.17632/859c935kb8.2
TMA_PCB	ZIP	1.0	CC BY 4.0	https://doi.org/10.17632/859c935kb8.2
TMA_Assem	ZIP	1.0	CC BY 4.0	https://doi.org/10.17632/859c935kb8.2
TMA_Source_code	ZIP	1.0	CC BY 4.0	https://doi.org/10.17632/859c935kb8.2

**Table 2:** Bill of mechanical materials for the TMA.

Designator	Component	Number	Cost per unit −currency	Total cost − currency	Source of materials	Material type
PD11001	Cover of the ring	2	$0.38	$0.76	[Custom supplier]	ABS plastic
PD11002	Ring basement	1	$0.91	$0.91	[Custom supplier]	ABS plastic
PD11004	Rounded head screw M2x5mm	4	$0.07	$0.28	https://www.mcmaster.com/products/screws/rounded-head-screws-2∼/length ∼ 5-mm/drive-style ∼ hex/	Metal
PD11005	Ring strap	1	$4.80	$4.80	https://mou.sr/4mSAJpy	Nylon
						
PD11007	Mainboard basement	1	$9.60	$9.60	[Custom supplier]	ABS plastic
						
PD11009	Cover of the mainboard basement	1	$3.53	$3.53	[Custom supplier]	ABS plastic
PD11010	Rounded head screw M3x5mm	4	$0.19	$0.76	https://www.mcmaster.com/products/screws/rounded-head-screws-2∼/length ∼ 5-mm/drive-style ∼ jis/	Metal
PD11011	Strap fixing	1	$1.32	$1.32	[Custom supplier]	ABS plastic
PD11012	Flat head screw M3x5mm	4	$0.05	$0.20	https://www.mcmaster.com/products/screws/flat-head-screws-2∼/metric-alloy-steel-hex-drive-flat-head-screws/thread-size ∼ m3/length ∼ 5-mm/	Metal
PD11013, PD11014, PD11015, PD11016,	Wrist strap	1	$7.5	$7.5	https://mou.sr/47RBWZK	Fabric
**Sum of cost**				**$29.66**

**Table 3:** Bill of electrical materials for the TMA.

Designator	Component	Number	Cost per unit −currency	Total cost − currency	Source of materials	Material type
USB-to-TTL modules	PL2303HX	1	$5.99	$5.99	https://a.co/d/dpF3ZI1 for	Semiconductors/others
SCL3300-D01-1	IMU sensor SCL3300	1	$39.35	$39.35	https://mou.sr/3UpqnBi	Polymer
R1, R2, R3, R4	Resistor 10 K	4	$0.1	$0.4	https://mou.sr/4llRLuM	Semiconductors/others
R5, R6, R7, R8, R1′	Resistor 470	5	$0.57	$2.85	https://mou.sr/45hyJkI	Semiconductors/others
C1, C2, C3, C4	10uF Capacitor	4	$0.23	$0.92	https://mou.sr/4mdNxqw	Semiconductors/others
C1′, C2′, C3′, C4′	100nF 25 V Capacitor	4	$0.12	$0.48	https://mou.sr/4fGRTE8	Semiconductors/others
J1′, J2, J3, J4	Headers/Wire Housings	4	$0.46	$1.84	https://mou.sr/412b4SI	Semiconductors/others
J5	Jumper 3	1	$2.1	$2.1	https://mou.sr/3Jajv8o	Semiconductors/others
J1	Headers/Wire Housings	1	$0.13	$0.13	https://mou.sr/3HZL8Ri	Semiconductors/others
LM1085IS-ADJ	Voltage Regulators	1	$2.57	$2.57	https://mou.sr/411gI7w	Semiconductors/others
ESP-12F	Microcontroller	1	$6.95	$6.95	https://mou.sr/45gNnsg	Semiconductors/others
BM06B-SRSS-TB(LF)(SN)		1	$0.9	$0.9	https://mou.sr/3Nyy0oX	Semiconductors/others
MOSI, MISO, CLK, 3 V3	Led 0805	5	$0.43	$2.15	https://mou.sr/3PxGf5d	Semiconductors/others
Reset	Button	1	$0.25	$0.25	https://mou.sr/45vLQ0a	Semiconductors/others
Signal wire	C-Grid Cable Assembly 26 AWG	1	$9.63	$9.63	https://mou.sr/41z7nUR	PVC plastic
**Sum of cost**				**$76.51**		

### 5 Build instructions

**Fig. 1:**
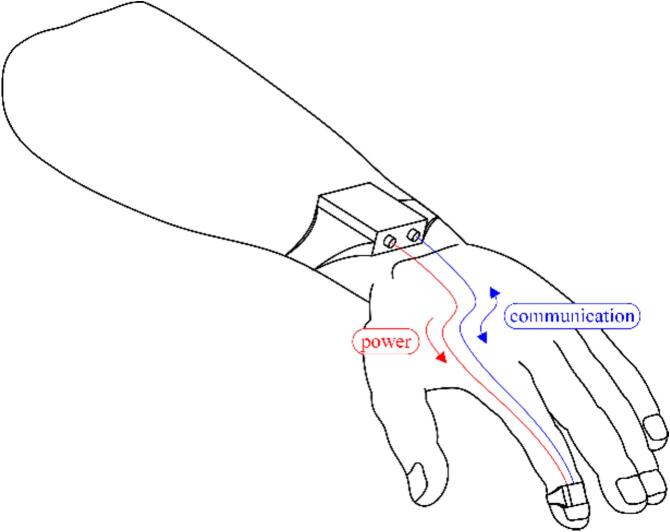
Schematic overview of the TMA.

Fig. 1 describes a schematic overview of TMA applied to the human upper limb. The wrist-wear component integrates a microcontroller unit and compact power supply, delivering both energy and a standardized communication protocol to the embedded IMUs, particularly SCL3300-D01-1, inside the wrist module. The two modules are connected by two lines: the red one represents the power wires transferring from the wrist module to the ring module, and the blue one demonstrates the signal wires for communicating between these modules. The design of TMA is flexible so that the ring module can be placed at any position along the length of each finger, not only at the top of the finger. This adjustable placement allows the sensor position to be varied during laboratory experiments, enabling students and researchers to examine how sensor location affects the measured acceleration signal. Cable length is selected based on a trade-off between signal integrity and mechanical flexibility in wearable device applications. The use of a longer cable is going to improve the ability to locate the wearables in space, but will increase propagation delay and also become more sensitive to noise and interference. In this prototype, the cable between the wrist and ring is 30 cm, giving rise to a propagation delay of 1.5 ns, based on an assumption of 5 ns/m for the signal delay [31]. The delay is inconsequential for a sensor operating at 40 Hz, and problems with signal integrity and susceptibility to EMI usually arise when the data communication rate is higher or longer interconnections [32].

### 5.1 Overall system description

**Fig. 2:**
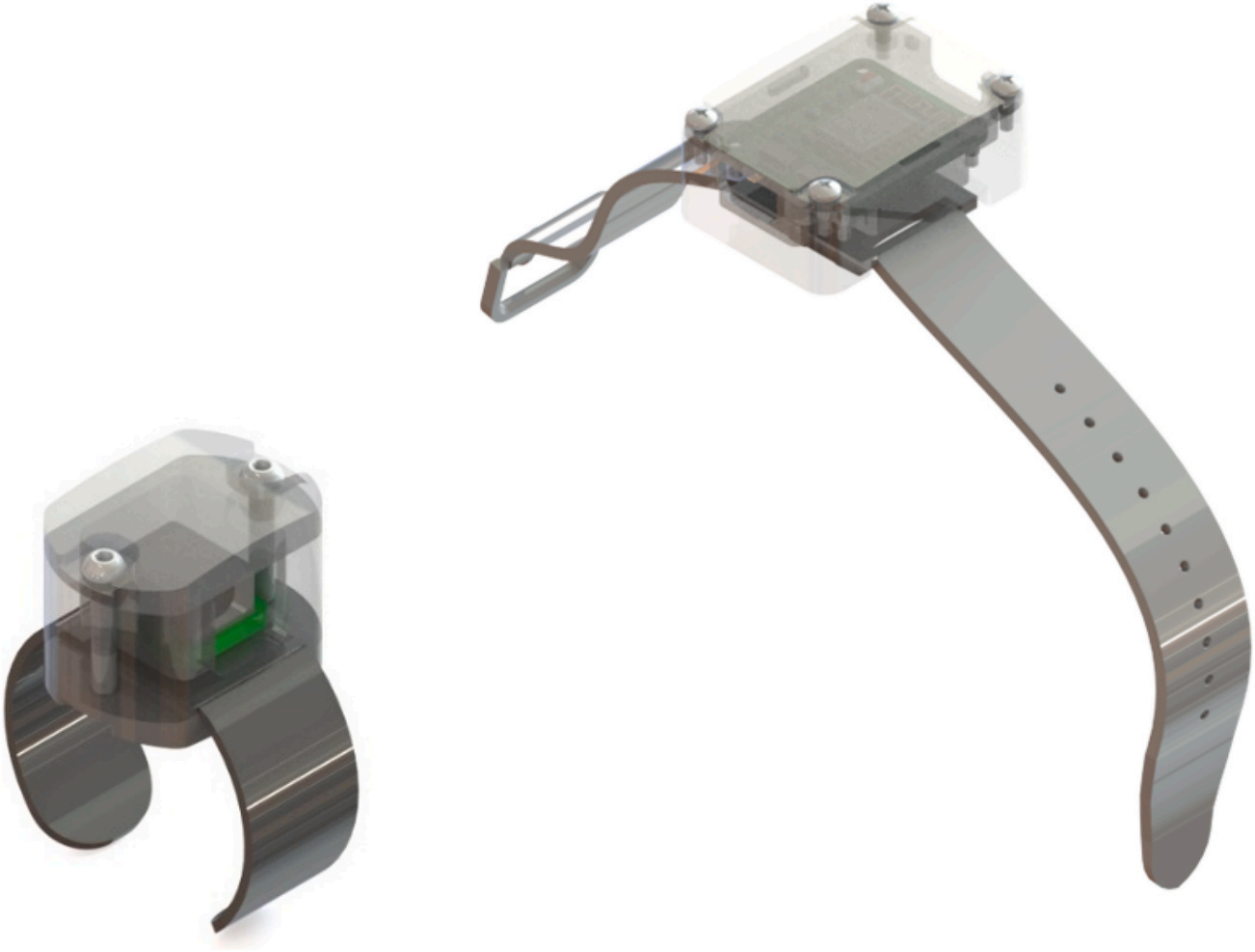
Transparent model of the wrist module and the ring module.

Fig. 2 displays a transparent model of the TMA, revealing the arrangement of key components within the TMA’s enclosure. The transparent housing exposes a suitable component layout, where both functional modules are strategically positioned along the forearm and finger’s longitudinal axis for anatomical conformity. A streamlined wiring harness connects the ring-embedded IMU to a centralized hub positioned at the ring base’s lateral aspect, which interfaces with the mainboard housed in the primary compartment via a front-mounted junction. Additionally, it is enhanced by the PCB and the IMU or the microcontroller integration, achieving spatially functional efficiency. Below, the ring and wrist strap are threaded, with the strap fixing way through so that it is worn on the finger and wrist.

### 5.2 Installation guide

**Fig. 3:**
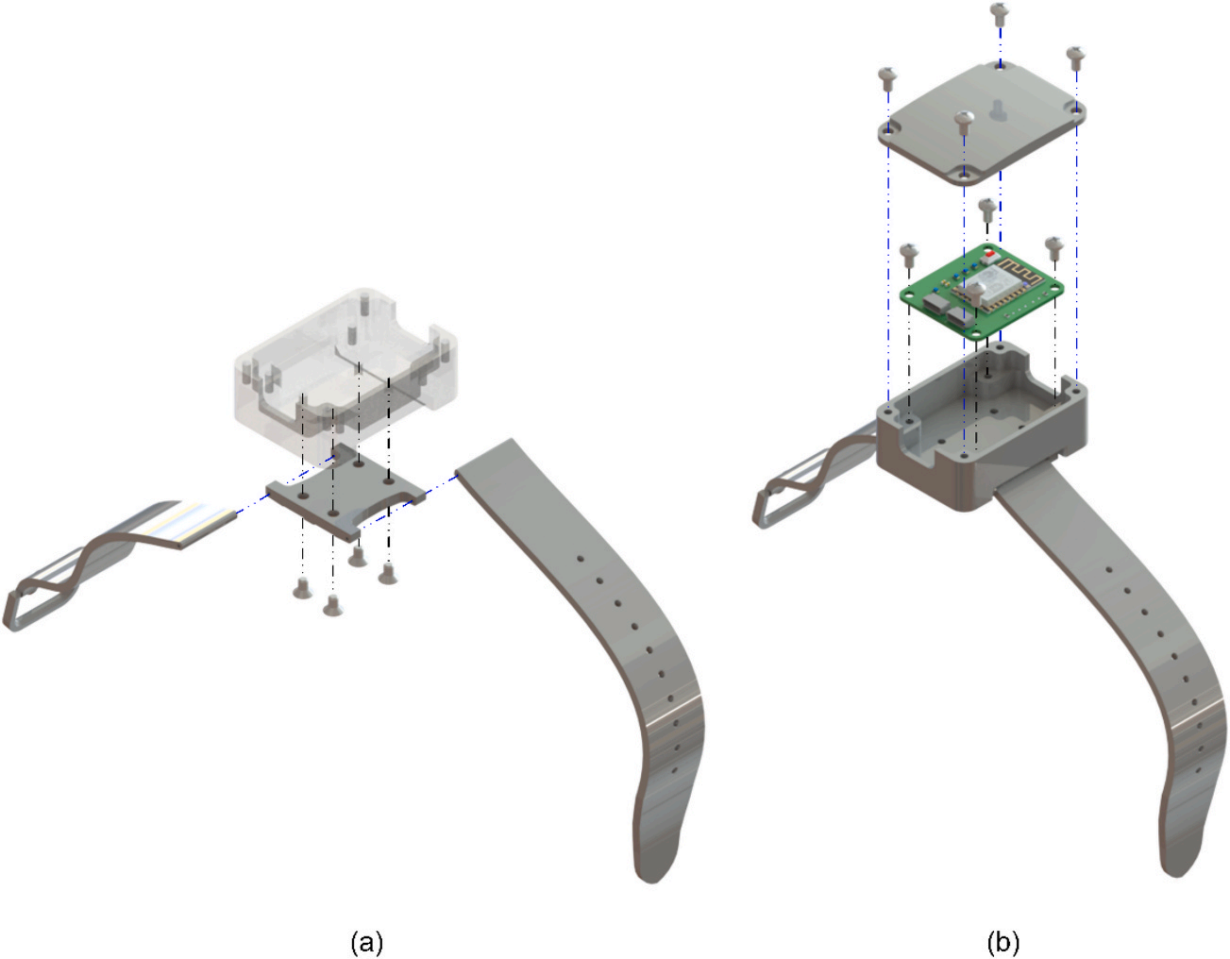
Installation of the wrist module for the TMA: (a) assembly of lower components, and (b) assembly of upper components.

Fig. 3 depicts the installation of the wrist-wear module, which includes two assembly stages. Firstly, the assembly begins with the foundational components, including the wrist strap and strap fixing, as shown in Fig. 3a. To ensure precise alignment, the lower section of the mainboard basement is designed with a C-shaped groove that fits the dimensions of the strap fixing. This design principle extends to the strap fixing surface, featuring an identical groove for seamless wrist strap attachment. By doing this, the mainboard basement, the strap fixing, and the strap can be guaranteed to share the same central plane and are fastened by using the flat head screws. This configuration increases structural stability and helps maintain an optimum balance for user comfort in prolonged use. The next step is the mounting of the main circuit board, illustrated in Fig. 3b. The inside of the mainboard is designed with a raised platform so that the mainboard can be mounted on it by using rounded head screws without damaging the mainboard’s surface below. The mainboard basement cover is affixed using additional rounded-head screws, shielding the internal electronics from external disturbances and completing the assembly.

**Fig. 4:**
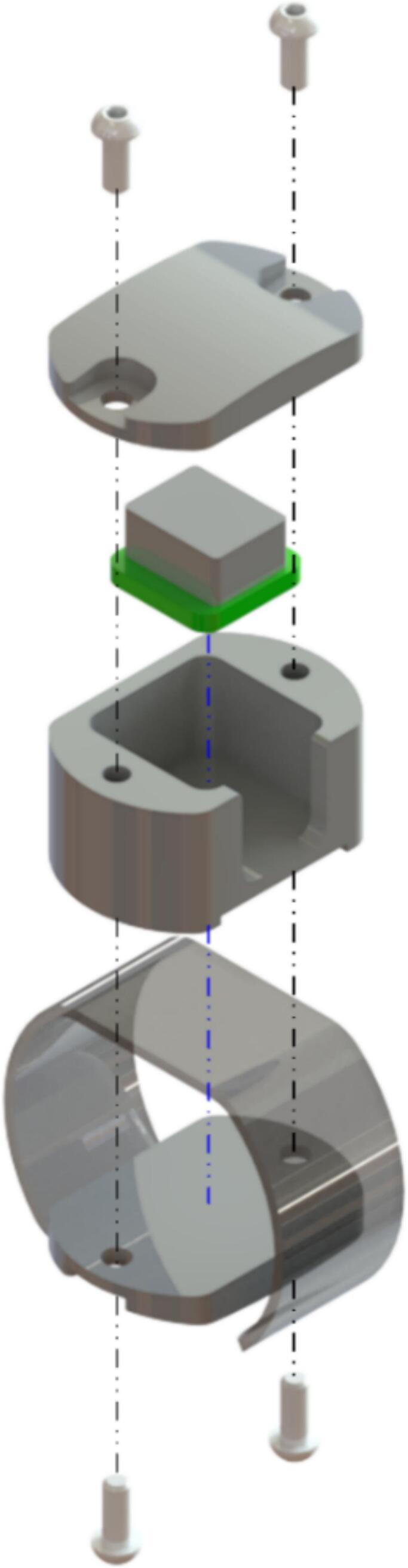
Installation of the ring module for the TMA.

Fig. 4 illustrates the installation of the ring-wear module. The same design idea of the mainboard basement is applied to the ring basement, in which the ring basement’s underside incorporates an identical C-channel groove fitting to the ring strap. One of the covers of the ring, the ring strap, and the ring base have the same central plane, secured by flat-head screws. The module’s compact design requires an innovative spatial optimization, in which the IMU nestles precisely within the ring’s interior cavity, eliminating the need for a secondary retention method. This approach of designing the self-retaining system ensures that the stability of the IMU is maintained while keeping the number of parts to a minimum. The assembly process is finished by attaching the other cover of the ring using round-head screws, which help isolate the electronics from any outside disturbance.

### 5.3 Internal wiring and component integration

**Fig. 5:**
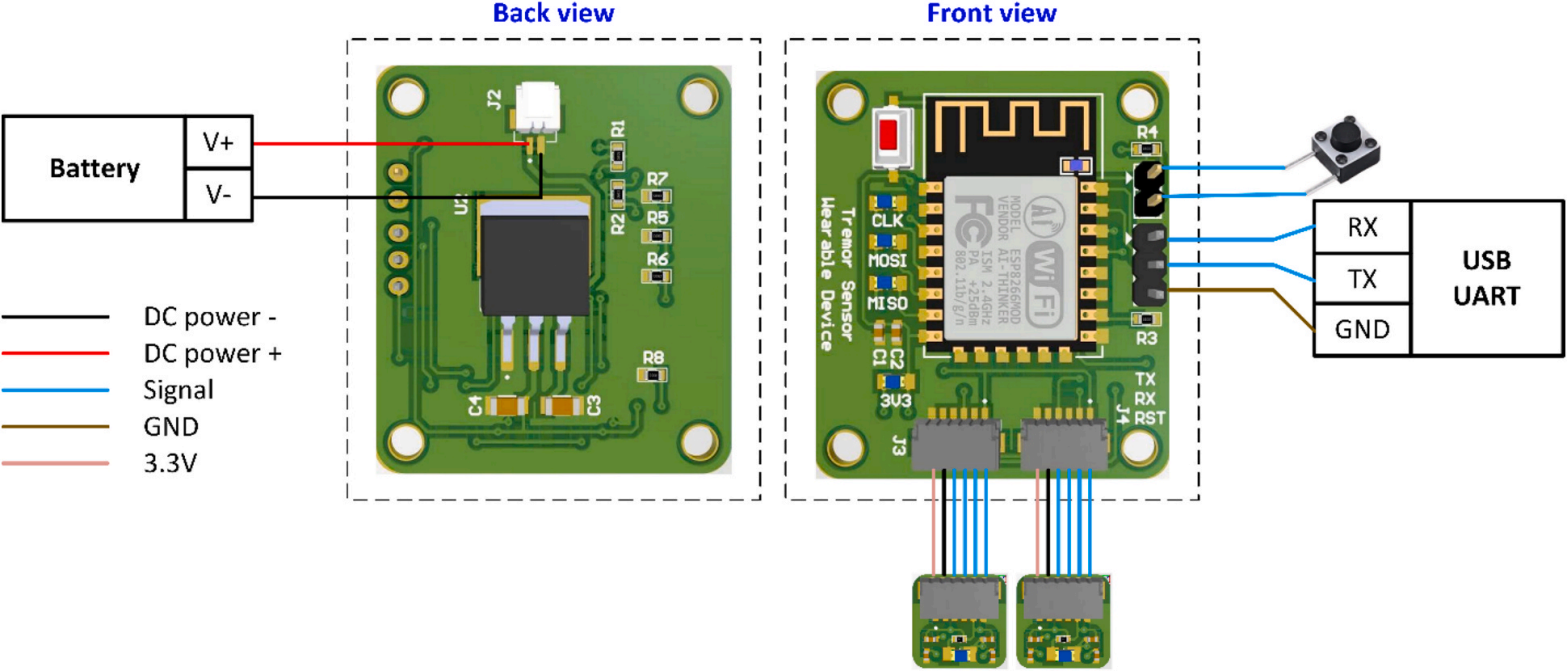
Diagram of interior wiring and components of TMA.

Fig. 5 illustrates the internal wiring and component integration of the wrist and ring modules. Initially, the 5VDC power supply is connected to the main controller board to supply power for the main controller and all connected components. Subsequently, the linear low-dropout regulator steps down from 5VDC to 3.3VDC for low-power peripherals, including IMUs and a microcontroller. In this architecture, the ESP-12F is mounted on the controller board as the microcontroller, as described in the electronic Bill of Materials, coordinating IMU data acquisition, data distribution, and data transmission to the storage station. Regarding the IMU, it is connected to the controller’s signal input and power supply, while communication with the main controller board is handled via SPI to enable high-speed full-duplex data streaming. Besides, the main controller board remains an interface for an extra IMU to simultaneously measure two fingers’ motion.

To quantify the power behavior of the TMA, the 3.3 V load current was estimated from the main active components under the maximum sensing configuration, where two SCL3300-D01-1 IMUs are operated simultaneously. According to the datasheets [33], [34] each SCL3300-D01-1 consumes approximately $I_{\mathrm{SCL}}=$ 2.1 mA in mode 4, while the ESP-12F module consumes approximately $I_{\mathrm{SCL}}=$ 170 mA during typical Wi-Fi transmission and requires a supply capable of supporting peak current above $I_{\mathrm{SCL}}=$ 500 mA. Therefore, considering two SCL3300-D01-1 IMUs, the total load current was estimated as $I_{\mathrm{load}}=I_{\mathrm{ESP}}+2I_{\mathrm{SCL}}$, giving 174.2 mA in typical Wi-Fi transmission and 504.2 mA in the peak-current condition. Since the LM1085RS-3.3 is a linear regulator, the ideal conversion efficiency $\left(\eta \right)$ and dissipated power $\left(P_{\mathrm{loss}}\right)$ are calculated as follows:

(2) $$\eta =\frac{V_{\mathrm{out}}}{V_{\mathrm{in}}}\times 100\%,P_{\mathrm{loss}}=\left(V_{\mathrm{in}}-V_{\mathrm{out}}\right)I_{\mathrm{load}},$$

where $V_{\mathrm{in}}$, $V_{\mathrm{out}}$, and $I_{\mathrm{load}}$ are the regulator input voltage, regulated output voltage, and total load current, respectively. By applying the regulator input voltage $V_{\mathrm{in}}=$ 5 V and regulated output voltage $V_{\mathrm{out}}=$ 3.3 V to Eq. (2), the ideal conversion efficiency of the LM1085RS-3.3 is estimated to be approximately 66%. Accordingly, the regulator dissipates approximately 0.296 W during typical Wi-Fi transmission and 0.857 W under the peak-current design condition, in which these values are analytical estimates derived from the nominal supply voltages and datasheet-based current consumption (without using thermocouple, infrared camera, or onboard temperature sensor). This result indicates that the LM1085RS-3.3 is the main heat-generating component, although the thermal load remains moderate during typical operation. This thermal aspect was considered in the mechanical design, where the 6 mm clearance on the regulator side leaves approximately 1 mm of free space above the LM1085RS-3.3 package, as shown in Fig. 3b. This clearance prevents direct contact between the regulator and the cover, while providing a small passive air gap that supports local heat spreading and reduces heat accumulation around the power IC during operation.

For firmware flashing, the USB to transistor-transistor logic (USB-to-TTL) module is applied to upload the program to the ESP-12F using the universal asynchronous receiver/transmitter (UART) communication. This converter circuit is only used when uploading the code and can be removed after finishing the uploading process. In addition, multiple communication protocols are incorporated to ensure data exchange among electric components. Finally, the converter circuit communicates with the host computer using TTL, ensuring compatibility. The detailed design and communication protocols are presented in TMA_Mainboard.PDF and TMA_PCB.ZIP.

### 5.4 Implementing a Blynk web interface for data monitoring

**Fig. 6:**
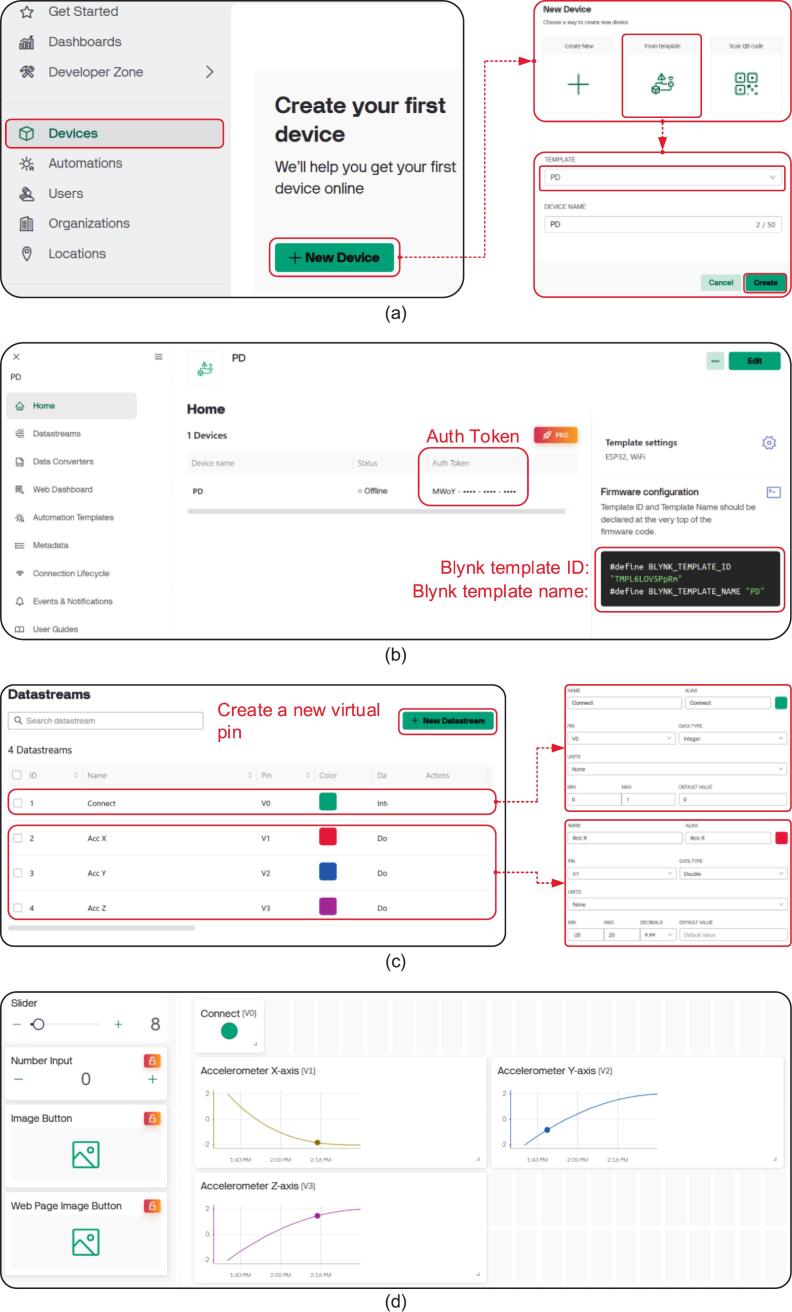
Configuration workflow of the Blynk-based IoT interface for real-time data visualization.

Within the framework of this study, the Blynk platform is utilized as an Internet of Things-based (IoT-based) visualization tool to monitor and display real-time data acquired from the IMUs. Fig. 6 illustrates the process of interface construction on the Blynk platform via the web console accessible at blynk.io/platform/web-console. Firstly, a new interface can be created by selecting ‘New Template’ under the ‘My Templates’ section of the ‘Developer Zone’. The project name needs to be declared, and the hardware type should be configured as ESP8266 with the connection type set to Wi-Fi. Subsequently, a ‘Device’ must be initialized by performing as Fig. 6a: selecting the ‘Devices’ tab, choosing ‘New Device’, selecting ‘From template’, locating the previously created template, and finally clicking ‘Create’. In the ‘Home’ tab of the newly created template, the 'Blynk Template ID’, ‘Blynk Template Name’, and ‘Auth Token’, as described in Fig. 6b, need to be recorded. These parameters are unique to the corresponding ‘Device’, and must be declared in the PD.ino program. In the ‘Datastream’ tab depicted in Fig. 6c, virtual pins need to be defined by selecting the ‘New Datastream’ tab. Specifically, virtual pin V0 is initialized with an integer data type to track the connection status between the device and Blynk. Similarly, the virtual pins (V1,…, Vn) are created with a double data type to display the acceleration data, with n being the number of data points from the IMU. Notably, the value range of the virtual pins (V1,…, Vn) is selected as [–5, 5], and ‘Decimals’ is in the form ‘#.##’ to observe 2 decimal places. In this study, virtual pins are defined in a way that corresponds to the data described in Table 4. Next, the interface is designed by dragging and dropping the ‘Widget Box’ into the ‘Dashboard’ box in the ‘Web Dashboard’ bar. In which the ‘LED’ block is used for V0, and the ‘Chart’ block is applied to the data pins (V1,…, Vn) as shown in Fig. 6d. Finally, the option ‘Save And Apply’ is clicked to complete the interface design process.

**Table 4:** List of virtual pins.

**Virtual pins**	**Data type**	**Widget box**	**Function**
V0	Integer	LED	Displays the device’s connection status to Blynk
V1	Double	Chart	Display acceleration data along the X-axis of IMU
V2	Double	Chart	Display acceleration data along the Y-axis of IMU
V3	Double	Chart	Display acceleration data along the Z-axis of IMU

## 6 Operation instructions

**Fig. 7:**
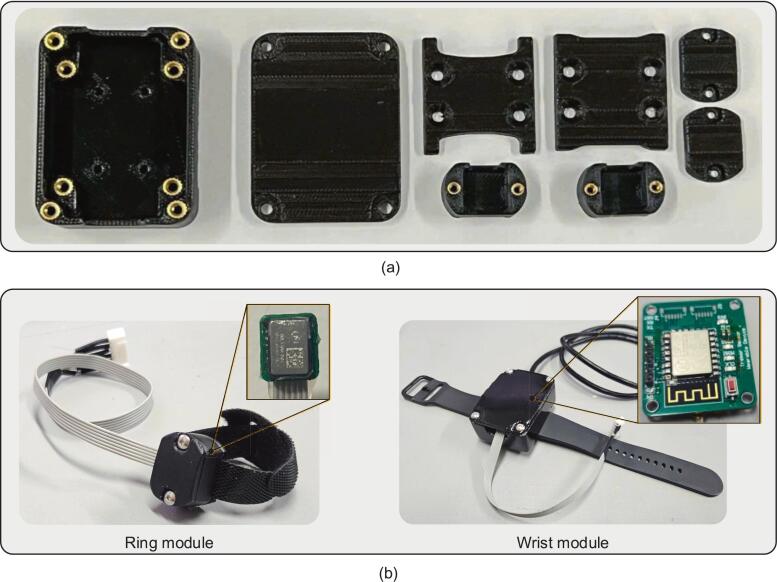
(a) Parts immediately after 3D printing, and (b) the fully assembled TMA with a refined surface.

Fig. 7 showcases the complete installation of TMA components after being manufactured using a 3D printing method and undergoing the surface refinement process by applying grinding and polishing techniques. The final product can overcome the drawbacks of the 3D printing method: first, they eliminate layer lines and micro-voids inherent to the printing process, creating an optimally smooth substrate for subsequent epoxy filler application and spray coating; second, they enhance the structural integrity of printed surfaces by reducing stress concentrators and improving impact resistance. For device operation, the ESP-12F microcontroller needs to be loaded with the PD.ino program using the Arduino IDE, which has pre-installed the libraries described in the TMA_Source_code folder listed in Table 1. It is important to mention that the button RST should first be pressed when powering up the system in order to ensure that the ESP is operating within the bootloader mode. In addition, the USB-to-TTL converter should be connected so that the converter's RX and TX pins are swapped with the corresponding pins of the device, while the GND of both components is commonly grounded. Once the firmware upload is successful, the RST button should be released, and the device should be rebooted for the booting process. In case the ESP lacks pre-configured Wi-Fi data in its flash memory, the ESP will automatically create an access point and the configuration portal to configure the network using any Wi-Fi-enabled device. On the contrary, the device can connect itself automatically, should there be any previously configured Wi-Fi network available. Besides, it is possible to reset Wi-Fi configurations by pressing and holding the RST button for over three seconds, which enables network modifications whenever required. Under operational conditions, a blinking behavior with a 1-second cycle can be detected using the LED widget. In case it fails to blink within one minute following Wi-Fi configuration, it means that the device has to be connected back to another wireless network using the RST button pressed for three seconds.

**Fig. 8:**
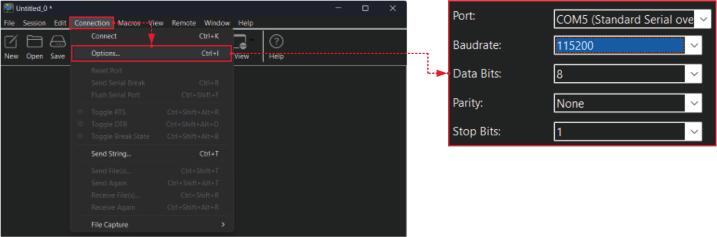
The process of setting communication parameters.

In this experiment, there were two ways used to observe data, which included wired using a USB to TTL module and wireless through Blynk. The wired method allows observing data with higher sampling accuracy, but the wireless method, through Blynk, allows observing remotely. CoolTerm software was employed for data visualization and logging in the USB-to-TTL circuitry. First of all, CoolTerm software needs to be configured to use the COM port assigned to the connected equipment, with communication parameters matching the PD.ino program, as shown in Fig. 8 (baud rate = 115200, data bits = 8, parity = ‘None’, stop bits = 1). The Connect tab will then be clicked on to begin the process of connecting until the status shows as “Connected” at the bottom of the screen. Lastly, data logging will take place using the keyboard keys Ctrl and R. The saved files from the experiment are going to be in CSV format. In the Blynk application, one needs to connect the device to a Wi-Fi network. Once the connection is established, data can be monitored by accessing blynk.cloud/dashboard/ and selecting the previously created ‘Device’.

**Fig. 9:**
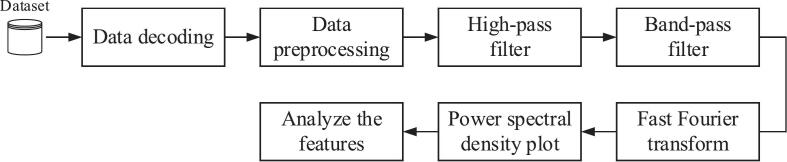
The flowchart of signal-processing pipeline.

Fig. 9 shows the signal-processing pipeline employed for the frequency-based analysis of the IMU signal related to the tremors. First, the raw data are decoded into timestamp information and three accelerometer channels. Relative time $t_{i}$ is obtained from the timestamp sequence by applying the expression $t_{i}=\tau_{i}-\tau_{0}$, wherein $\tau_{i}$ is the i-th sample’s timestamp, whereas $\tau_{0}$ is the initial timestamp in the sequence. Following this, invalid entries in the timestamp sequence are ignored, whereas other samples are placed in chronological order to preserve the acquisition sequence. The sampling interval is calculated as $\Delta t_{i}=t_{i}-t_{i-1}$, and the representative interval is estimated as $\Delta t_{med}=median\left(\Delta t_{i}\right)$. To compensate for intermittent transmission gaps, abnormal intervals are detected when $\Delta t_{i}>6\Delta t_{med}$, and the corrected time base is reconstructed as

(3) $${\tilde{t}}_{i}=t_{i}-\sum_{k<i,k\in G}e_{k},$$

where G is the set of detected gap indices and $e_{k}=max\left(\Delta t_{k}-\Delta t_{med},0\right)$ is the excess delay associated with the k-th gap. After preprocessing, the cleaned acceleration data are represented by the three-axis vector $a\left({\tilde{t}}_{i}\right)={\left[a_{x}\left({\tilde{t}}_{i}\right),a_{y}\left({\tilde{t}}_{i}\right),a_{z}\left({\tilde{t}}_{i}\right)\right]}^{T}$, and the effective sampling frequency is estimated as

(4) $$f_{s}=\frac{1}{median\left({\tilde{t}}_{i}-{\tilde{t}}_{i-1}\right)}.$$

To reduce orientation dependency, the scalar acceleration magnitude, defined by the Euclidean norm of the triaxial acceleration vector $a_{m}\left({\tilde{t}}_{i}\right)={\|a\left({\tilde{t}}_{i}\right)\|}_{2}$, is considered instead of individual acceleration components. In the high-pass and band-pass filtering steps, the scalar acceleration magnitude is sequentially processed to remove non-oscillatory components and isolate the tremor-related frequency range. Specifically, the high-pass Butterworth filter is configured with a cutoff frequency of 0.5 Hz, whereas the band-pass Butterworth filter [35] is configured to retain components from 4 Hz to 12 Hz. Both filters can be expressed using the general digital filter form

(5) $$y\left[n\right]=\sum_{r=0}^{M}b_{r}x\left[n-r\right]-\sum_{s=1}^{N}a_{s}y\left[n-s\right],$$

where x[n] and y[n] are the input and output signals of each filtering stage, respectively. The coefficients $b_{r}$ and $a_{s}$ are the feedforward and feedback coefficients of the Butterworth filter, while M and N denote the corresponding feedforward and feedback filter orders. After filtering, the signal is divided into 10 s analysis windows with 50% overlap. In the fast Fourier transform and PSD estimation step, the output of the band-pass filter is analyzed in each window using Welch’s method [36] to quantify the signal power distribution over frequency. Specifically, each filtered segment is multiplied by a Hamming window before applying the FFT, which is expressed as

(6) $$X_{m}\left(f\right)=\sum_{n=0}^{N}x_{m}\left[n\right]w\left[n\right]e^{-\frac{j2\pi fn}{f_{s}}},$$

where $x_{m}\left[n\right]$ is the m-th filtered signal segment, $N_{w}$ is the number of samples in each analysis window, $f_{s}$ is the sampling frequency, and f is the frequency variable. The Hamming window is defined as

(7) $$w\left[n\right]=0.54-0.46\cos\left(\frac{2\pi n}{N_{w}-1}\right),$$

Based on the $w\left[n\right]$ and $X_{m}\left(f\right)$, the Welch PSD estimate for the m-th window is then calculated as

(8) $$P_{m}\left(f\right)=\frac{1}{f_{s}U}{\left|X_{m}\left(f\right)\right|}^{2},$$

where $U=\sum_{n=0}^{N_{w}-1}w^{2}\left[n\right]$ is the Hamming-window normalization factor. In view of the PSD results, the dominant spectral peak is studied to determine the major oscillatory frequency in the measured acceleration signal, thereby confirming the presence of tremor-like components in the data.

## 7 Validation and characterization

### 7.1 Experimental setup

**Fig. 10:**
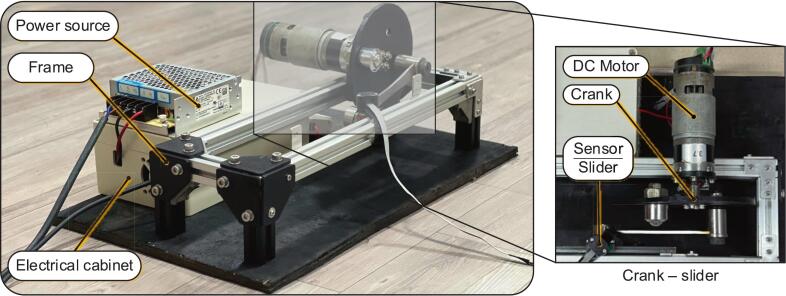
Overview of the experimental system.

Fig. 10 below shows the setup used in measuring acceleration using the TMA sensor. The setup uses a slider crank mechanism that consists of four major components: the frame, power source, control box, and slider-crank assembly. The IMU, especially the SCL3300-D01-1 model, is attached to the slider, which is actuated via the crank mechanism connected to the DC motor having a 1:3.7 gear system. Additionally, the orientation of the IMU is such that the Z-axis is vertical, whereas the X and Y axes make an angle of 45° with the sliding direction. The frame provides structural stability during operation, while the electrical cabinet and power source perform computation and deliver control signals to the DC motor. In this case, the Arduino Nano development board is applied to implement the control algorithm, and an encoder with 13 pulse resolution observes the feedback angular velocity of the DC motor. Through the slider-crank mechanism, the slider exhibits harmonic motion with an amplitude of 40 mm and a frequency of 30 ω/π, where ω denotes the angular velocity of the DC motor in rpm. In this case, the purpose of using the slider-crank mechanism was to create a mechanical tremor source, aiming to evaluate acceleration acquisition and dominant oscillation-frequency estimation of the TMA device under repeatable laboratory conditions. To avoid any misunderstanding, it is emphasized that the slider-crank mechanism fails to be employed as a validated surrogate of Parkinsonian tremor and reproduce the full biomechanical, voluntary, or non-stationary characteristics of human pathological tremor.

**Fig. 11:**
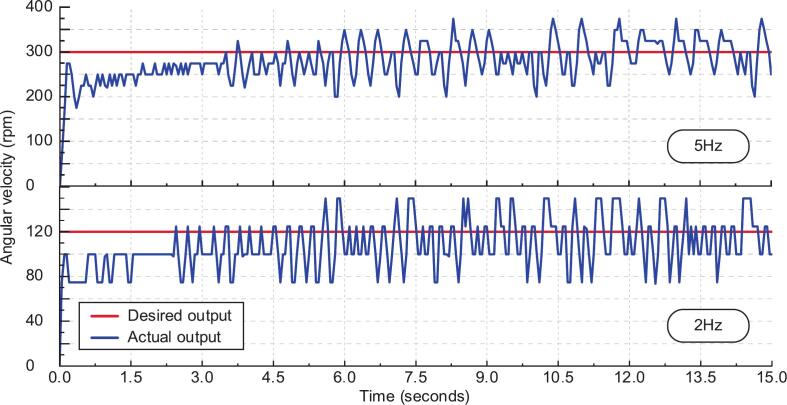
Angular velocity response of the DC motor in a 2 Hz and 5 Hz frequency scenario.

Two slider motion scenarios were considered in this study, corresponding to amplitudes of 40 mm at frequencies of 2 Hz and 5 Hz. Accordingly, the angular velocity of the DC motor was controlled at 120 rpm for 2 Hz and 300 rpm for 5 Hz. The maximum steady-state errors were recorded as 24.74% (2 Hz) and 33.47% (5 Hz), respectively, with the corresponding responses illustrated in Fig. 11. For the proposed device, the sampling interval in the wired communication mode was set to 0.025 s by assigning the variable READ_PERIOD_MS a value of 25 in the PD.ino file. In the Blynk-based observation mode, the sampling interval was increased to 0.2 s to comply with the platform’s minimum configuration, achieved by setting SEND_PERIOD_MS to 200 in the PD.ino file.

### 7.2 Validation result and characteristics

**Fig. 12:**
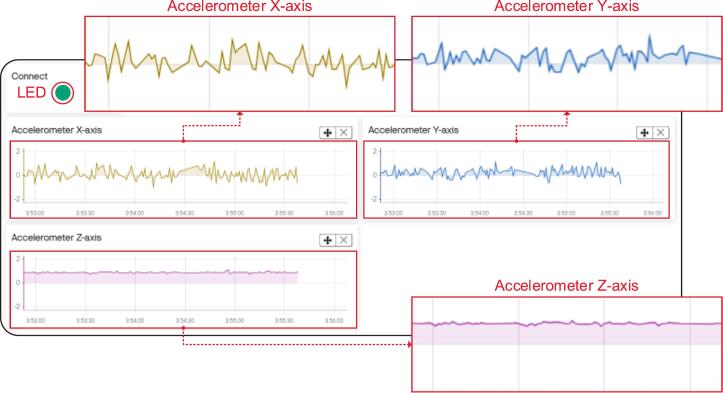
Data monitoring dashboard of the Blynk platform.

Considering the relatively low sampling frequency of the Blynk interface, an experimental scenario with an oscillation frequency of 2 Hz was selected to validate this observation method. This experiment aims to verify the device’s ability to detect oscillatory motion and extract dominant frequency components under controlled mechanical excitation. Fig. 12 presents the data monitoring dashboard based on the Blynk platform, which displays four parameters: the connection status and the three-axis acceleration graphs. The data are visualized in real time with an update period of 0.2 s and are temporarily stored on the Blynk cloud from the moment the web dashboard is accessed. Notably, data can be lost upon disconnection or page reload within the Blynk web interface for non-paid accounts. In addition, the graphs reveal several intervals where the variations in acceleration along the three axes are negligible, even though the system operates nominally at a frequency of 2 Hz. This observation indicates potential data loss or transmission latency during communication with the Blynk cloud. The phenomenon is attributed to unstable Wi-Fi conditions or limited network bandwidth, which may cause temporary interruptions in the data-upload process.

Fig. 12 illustrates the acceleration signals recorded under the 5 Hz oscillation scenario using the wired acquisition method. The results demonstrate that data loss and transmission delays are effectively eliminated, in contrast to the case observed with the Blynk interface. The acceleration curves exhibit strong oscillatory behavior with repeating large-amplitude variations, consistent with the near-harmonic motion of the slider. In both experimental scenarios, the acceleration along the Z-axis remains approximately 1 g (where g=9.81m/sÂ²), while the X- and Y-axis accelerations fluctuate around zero. This is explained by the SCL3300-D01-1 orientation, where the Z-axis is aligned with gravity, maintaining a constant reading near 1 g, while the X and Y components vary according to the slider’s motion. The plots reveal several instances where the Z-axis acceleration approaches zero, even though the Z-axis of the SCL3300-D01-1 consistently points downward. This behavior indicates the presence of outliers in the dataset, suggesting that preprocessing is required before further analysis.

**Fig. 13:**
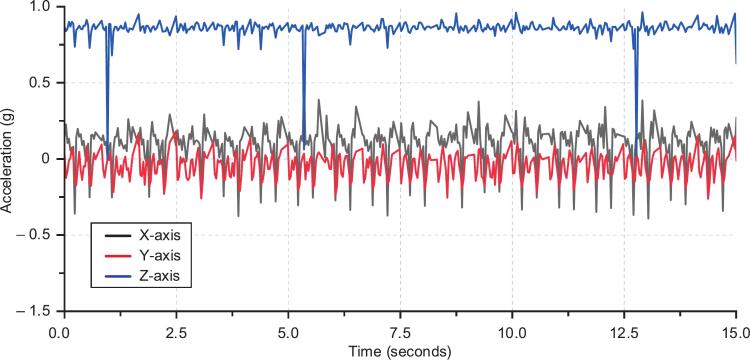
Measured acceleration data under 5 Hz mechanical excitation (wired acquisition mode, fs = 40 Hz).

**Fig. 14:**
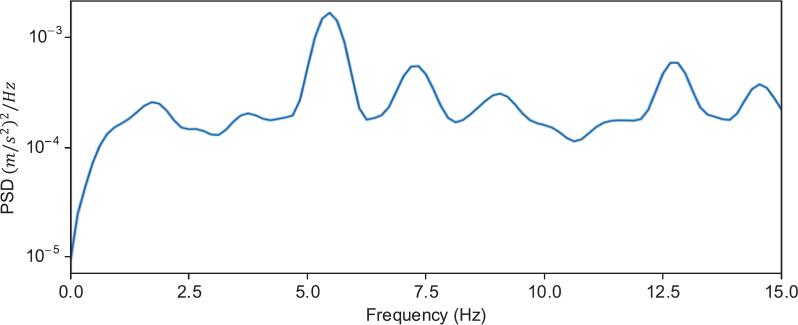
Power spectral density of filtered acceleration signal under controlled oscillatory excitation at 5 Hz.

To verify the device’s ability to detect oscillatory motion, the dataset shown in Fig. 13 was analyzed in the frequency domain using PSD estimation. Fig. 14 presents the PSD of the filtered total acceleration signal, where the main spectral peak represents the dominant oscillatory component captured by the TMA. The main spectral peak appears at 5.5 Hz, which deviates by 0.5 Hz from the intended excitation frequency. Additional peaks are observed at 7.5 Hz, 9 Hz, and 13 Hz, indicating the presence of higher-order oscillations. These frequency components are attributed to fluctuations in the angular velocity response of the DC motor during operation.

**Fig. 15:**
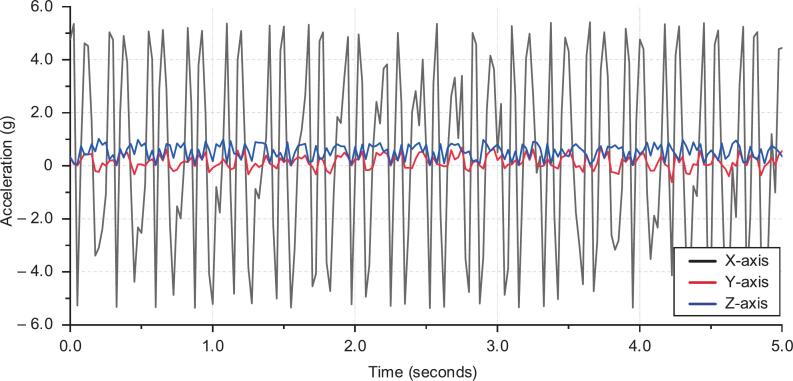
Measured acceleration data under 8 Hz mechanical excitation (wired acquisition mode, fs = 40 Hz).

To further investigate the response of the proposed device under a different oscillatory condition, an additional experiment was conducted using the same slider–crank mechanism with a nominal excitation frequency of 8 Hz. The measured acceleration signals along the three axes are shown in Fig. 15 as time-domain responses. Accordingly, the measured signals exhibit a clear oscillatory pattern throughout the observation interval, confirming that the proposed device is able to capture the periodic motion generated by the test rig. The X-axis acceleration shows the largest oscillation amplitude, whereas the Y-axis and Z-axis components remain at lower levels. This behavior indicates that the dominant motion of the mechanical excitation is mainly aligned with the X-axis of the sensor, while the remaining axes contain secondary vibration components and cross-axis coupling effects. In addition, the waveform deviates from a purely sinusoidal form, and several irregular peaks can be observed, suggesting that the excitation is affected by speed fluctuations of the DC motor and by nonlinear transmission effects of the slider–crank mechanism.

**Fig. 16:**
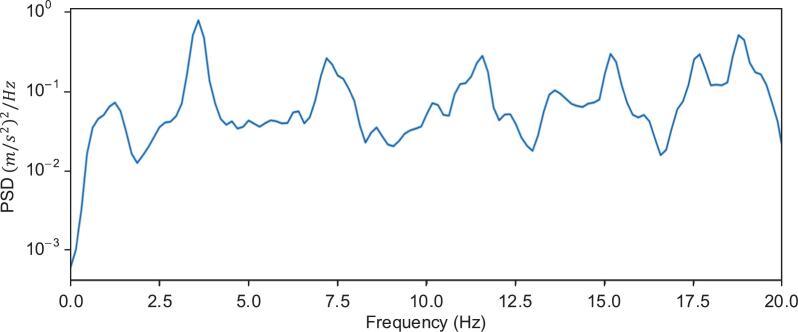
Power spectral density of filtered acceleration signal under controlled oscillatory excitation at 8 Hz.

To examine the spectral characteristics of the measured response, the 8 Hz dataset was analyzed in the frequency domain using Welch PSD estimation. As shown in Fig. 16, the PSD exhibits multiple spectral components rather than a single dominant peak, indicating the presence of harmonic or parasitic components from the non-ideal excitation system. Although the spectral distribution is more complex, a clear component remains present within the 4–––12 Hz tremor band. The result indicates that the estimated peak frequencies $f_{p}$ obtained from the spectral analysis range between approximately 5.5 Hz and 11.5 Hz, with a median value of about 7.34 Hz. The corresponding spectral peak width is approximately 1.09 Hz, indicating that the oscillatory component is concentrated within a relatively narrow frequency band. These results demonstrate that the proposed system can consistently identify oscillatory motion within the tremor frequency band even when the excitation signal contains multiple spectral components and mechanical disturbances.

To further evaluate the measurement accuracy and repeatability, the acceleration signal was segmented into overlapping analysis windows, and the dominant frequency $f_{i}$ within the tremor band was extracted from each window. The mean detected frequency is calculated as

(9) $$\overset{¯}{f}=\frac{1}{N}\sum_{i=1}^{N}f_{i},$$

where N denotes the number of analysis windows. The variability of the detected frequencies is characterized by the standard deviation, calculated by

(10) $$\sigma_{f}=\sqrt{\frac{1}{N-1}\sum_{i=1}^{N}{\left(f_{i}-\overset{¯}{f}\right)}^{2}}.$$

To quantify the deviation between the detected frequencies and the nominal excitation frequency $f_{\mathrm{ref}}$, the mean absolute error (MAE) and root mean square error (RMSE) are computed as

(11) $$\mathrm{MAE}=\frac{1}{N}\sum_{i=1}^{N}\left|f_{i}-f_{\mathrm{ref}}\right|,RMSE=\sqrt{\frac{1}{N}\sum_{i=1}^{N}{\left(f_{i}-f_{\mathrm{ref}}\right)}^{2}}.$$

To describe the uncertainty of the window-based frequency estimation, the expanded uncertainty of the mean detected frequency was calculated from the standard error of the detected frequencies. According to the general uncertainty-expression framework, the expanded uncertainty is obtained by multiplying the standard uncertainty by a coverage factor [37]. Therefore, the standard uncertainty and expanded uncertainty are calculated as

(12) $$u_{\overset{¯}{f}}=\frac{\sigma_{f}}{\sqrt{N}},U_{f}=t_{0.975,N-1}u_{\overset{¯}{f}}$$

where $u_{\overset{¯}{f}}$ is the standard uncertainty of the mean detected frequency, $U_{f}$ is the expanded uncertainty at approximately 95% confidence, and $t_{0.975,N-1}$ is the Student’s t-coverage factor. The detected frequency is then reported as $\overset{¯}{f}\pm U_{f}$, while MAE and RMSE are retained as error metrics relative to the nominal excitation frequency.

Based on Eqs. (9)-(12), the statistical analysis of the 8 Hz excitation experiment yields an average detected frequency of approximately 7.27 ± 0.14 Hz at approximately 95% confidence, with a standard deviation of 0.09 Hz during the stable portion of the signal. Additionally, MAE and RMSE are calculated as 0.73 Hz and 0.74 Hz, respectively. These results indicate that the dominant oscillatory component remains close to the intended excitation frequency, while the small standard deviation and expanded uncertainty indicate low variability of the detected dominant frequency across analysis windows. For the 5 Hz excitation experiment, the detected frequencies exhibit a larger dispersion due to fluctuations in the mechanical excitation system. The estimated mean frequency is 6.78 ± 1.07 Hz at approximately 95% confidence, with a standard deviation of 2.71 Hz, while the corresponding MAE and RMSE are 2.23 Hz and 3.20 Hz, respectively. The relatively large error and dispersion were associated with several factors. First, the nominal frequency was derived from the commanded motor speed rather than from an independent high-resolution measurement of the actual slider motion. As shown by the motor-speed response, the test rig exhibited substantial steady-state error and temporal speed fluctuations. Second, the nonlinear kinematics and mechanical clearances of the slider-crank mechanism introduced harmonic and parasitic components that could become larger than the fundamental component within individual analysis windows. Third, the 40 Hz sampling rate, finite window length, data outliers, and peak-selection procedure limited spectral resolution and could cause the dominant-frequency estimator to select a harmonic rather than the intended fundamental frequency. Consequently, the present frequency results primarily characterize the combined response of the prototype, the acquisition chain, the signal-processing algorithm, and the non-ideal mechanical excitation system. Briefly, the quantitative evaluation shows that the proposed device is capable of consistently detecting oscillatory motion within the tremor frequency band even under non-ideal excitation conditions.

### 7.3 Conclusion

In this research, a low-cost wearable motion-sensing prototype has been designed and evaluated. Translational acceleration in three directions is measured by the IMU sensors, specifically SCL3300-D01-1, in combination with the ESP-12F controller. Experimental results show that the TMA's performance for periodic motion detection and analysis is satisfactory in both wired and wireless modes. With the wired setup, stable sampling of 0.025 s was attained, with negligible latency, enabling precise documentation of the acceleration signal, which was an indication of the harmonic motion of the slider-crank system. The wireless configuration was based on the Blynk app that allowed the system to monitor and visualize data remotely using a web interface. Additionally, further experiments performed at 5 Hz and 8 Hz mechanical oscillation also proved that the system can reliably detect oscillatory movement within the tremor frequency range. Quantitative evaluation using window-based spectral analysis showed repeatable frequency estimates with MAE and RMSE values. Despite the non-ideal behavior of the mechanical excitation system, these results indicate that the device can capture oscillatory content, but further calibration and validation against an independently measured reference are required. In terms of safety, comfort, and usability aspects, the TMA was developed as a non-invasive prototype intended for short-term experimental and demonstrational purposes. Neither the wrist module nor the finger module penetrates any tissue, as no pressure-based fixation is implemented in the design. A total weight of about 115 g was chosen to avoid mechanical load on the hand and wrist in short-duration experiments. The exterior of the enclosure was rounded to minimize sharp edges in construction, providing greater comfort when worn. From an electrical point of view, power supply voltage of the device is limited by 5 V input and internal 3.3 V regulation of electronic circuitry in order to lower the electrical hazard. The enclosure also provides isolation between the circuit board and the user's body. It should be noted that neither ergonomic testing nor a skin irritation study was done in this work. Overall, the TMA achieved a good balance between accuracy, simplicity, and cost-effectiveness, making it suitable for laboratory experiments, educational purposes, and a prototype for applications in tremor-like motion acquisition and analysis.

In spite of all these positive findings, there have been a number of issues encountered in this experiment. The wireless transfer mode showed some data loss and delay while being performed in poor Wi-Fi connection environments. There were also some outliers found in the acceleration signal, which might have been generated due to electrical interference or an IMU drifting effect. Consequently, some filtering is recommended prior to conducting any frequency analysis. Moreover, there were no experiments with human subjects included in this project yet.

Future efforts shall include making the TMA robust and autonomous. The inclusion of onboard storage and buffering would ensure that there are no losses in case of network failures. Using an alternative and lightweight communication protocol such as MQTT would make it possible to transmit the collected data more reliably. The inclusion of adaptive filtering and calibration would help increase accuracy and repeatability. This way, the TMA would serve as a flexible and scalable sensor platform for analyzing tremors. Besides, in order to apply the device on the practical daily-life motion, extending the algorithm to differentiate pathological tremor from voluntary or daily-life movements is focused on, which enables separation of tremor oscillations from background motor activity, as well as conduct experiments on involving Parkinson's patients.

Besides, from a research and education platform perspective, this TMA will be incorporated in the laboratory models and experimental apparatus to provide an easier understanding of sensors, measuring instruments, embedded programming, communications, and data processing. Specifically, regarding engineering students, the TMA is going to incorporate laboratory models as well as real experiment designs for a more intuitive learning experience on sensors, measurements, embedded programming, and communication processes. As mentioned above, since the circuit board and software are self-designed, students can adjust the sensor interface, sampling parameters, communication protocols, and processing function according to different learning purposes. Moreover, TMA can also be applied as a sensory data acquisition component to gather the actual signals for subjects like sensors and measurement, embedded systems, digital signal processing, Internet of Things, and engineering data analysis. Based on that, it gives students the chance to actually apply skills like filtering, noise reduction, frequency analysis, feature extraction, and visualization to real-world data as opposed to just theoretical or simulated data. As a result, the platform would facilitate project-based learning by linking problem formulation, system design, experimentation, data analysis, and continuous improvement within a complete engineering process, which is often difficult to achieve through traditional teaching methods alone.

## Declaration of generative AI use

The authors used generative AI tools solely for language editing and to improve the clarity and readability of the manuscript. No AI tools were used to generate scientific content, perform data analysis, or influence the interpretation of results. All ideas, methods, analyses, and conclusions are the sole responsibility of the authors.

## Ethics statement

This study did not involve clinical validation, which means that there is no human participants, human biological samples, personal health information, or animals.

## CRediT authorship contribution statement

**Nhut Thang Le:** Writing – original draft, Resources, Methodology, Investigation, Conceptualization. **Ngoc Hung Nguyen:** Visualization, Software, Data curation. **Cong Toai Truong:** Software, Investigation, Data curation, Conceptualization. **Huy Hung Nguyen:** Validation, Project administration, Formal analysis. **Tan Tien Nguyen:** Supervision, Funding acquisition. **Van Tu Duong:** Writing – review & editing, Methodology, Conceptualization.

## Declaration of competing interest

The authors declare that they have no known competing financial interests or personal relationships that could have appeared to influence the work reported in this paper.
